# The Effects of Vitamin D Supplementation before 20 Weeks of Gestation on Preeclampsia: A Systematic Review

**DOI:** 10.3390/jpm13060996

**Published:** 2023-06-14

**Authors:** George Dahma, Gowry Reddy, Marius Craina, Catalin Dumitru, Alin Popescu, Lavinia Stelea, Radu Neamtu, Adrian Gluhovschi, Razvan Nitu, Anca Laura Maghiari, Gianina Tapalaga, Diana Aurora Arnautu, Aditya Nelluri, Ram Kiran Maganti, Elena Bernad

**Affiliations:** 1 Department of Obstetrics and Gynecology, Faculty of General Medicine, “Victor Babes” University of Medicine and Pharmacy Timisoara, 300041 Timisoara, Romania; george.dahma@umft.ro (G.D.); mariuscraina@hotmail.com (M.C.); dumitru.catalin@umft.ro (C.D.); popescu.alin@umft.ro (A.P.); radu.neamtu@umft.ro (R.N.); adigluhovschi@yahoo.com (A.G.); nitu.dumitru@umft.ro (R.N.); ebernad@yahoo.com (E.B.); 2 Doctoral School, Faculty of General Medicine, “Victor Babes” University of Medicine and Pharmacy Timisoara, 300041 Timisoara, Romania; 3 New York Medical College at St. Mary’s and St. Clare’s Hospital, Denville, NJ 07834, USA; greddy3@primehealthcare.com; 4 Clinic of Obstetrics and Gynecology, “Pius Brinzeu” County Clinical Emergency Hospital, 300723 Timisoara, Romania; 5 Center for Laparoscopy, Laparoscopic Surgery and In Vitro Fertilization, “Victor Babes” University of Medicine and Pharmacy Timisoara, 300041 Timisoara, Romania; 6 Department of Anatomy and Embryology, “Victor Babes” University of Medicine and Pharmacy Timisoara, 300041 Timisoara, Romania; boscu.anca@umft.ro; 7 Department of Odontotherapy and Endodontics, Faculty of Dental Medicine, “Victor Babes” University of Medicine and Pharmacy Timisoara, 300041 Timisoara, Romania; tapalaga.gianina@umft.ro; 8 Department of Cardiology, “Victor Babes” University of Medicine and Pharmacy Timisoara, 300041 Timisoara, Romania; aurora.bordejevic@umft.ro; 9 School of General Medicine, Sri Siddhartha Medical College, Tumakuru 572107, India; nelluriaditya@gmail.com; 10 School of General Medicine, Sri Devaraj Urs Academy of Higher Education and Research, Tamaka, Kolar 563101, India; ramkiran.maganti11@gmail.com

**Keywords:** vitamin D, vitamin D deficiency, pregnancy complications, gestational hypertension, preeclampsia

## Abstract

Preeclampsia is a leading cause of maternal and fetal morbidity and mortality worldwide. The role of vitamin D supplementation during early pregnancy in the prevention of preeclampsia remains unclear. Our objective was to synthesize and critically appraise the available evidence from observational and interventional studies to determine the effects of early pregnancy vitamin D supplementation on the risk of preeclampsia. A systematic review was conducted in March 2023 using PubMed, Web of Science, Cochrane, and Scopus databases, including literature published up to February 2023. In adherence to PRISMA guidelines, a structured and systematic search strategy was employed. A total of five studies were included in the review, encompassing 1474 patients. Overall, vitamin D supplementation during early pregnancy was associated with a reduced incidence of preeclampsia in all studies (ORs ranging from 0.26 to 0.31), while others showed an increased risk of preeclampsia with low vitamin D levels during the first trimester (ORs of 4.60, 1.94, and 2.52). However, other studies found no significant protective effect but good overall safety for various vitamin D dosages administered during the first trimester. Nevertheless, variations in vitamin D dosage, the timing of supplementation, and definitions of vitamin D insufficiency may have contributed to the inconsistencies in the observed outcomes. Some studies reported significant secondary outcomes, such as a reduction in blood pressure, preterm labor, and improved neonatal outcomes, such as birth weight. The evidence from this systematic review suggests that early pregnancy vitamin D supplementation may have a role in reducing the risk of preeclampsia. However, inconsistencies in the timing of supplementation, dosages, and methodological differences between studies highlight the need for further research to determine the optimal supplementation strategy and to clarify the relationship between vitamin D and preeclampsia risk.

## 1. Introduction

Preeclampsia is a multisystem disorder affecting approximately 2–8% of pregnancies globally, and it poses significant risks to both maternal and fetal health [1,2]. This condition is characterized by new-onset hypertension and proteinuria after 20 weeks of gestation and may present with or without accompanying systemic organ dysfunction [3,4]. Preeclampsia contributes to maternal morbidity and mortality, as well as adverse perinatal outcomes such as preterm birth, intrauterine growth restriction, and neonatal mortality [5]. Despite advancements in obstetric care, the etiology and pathophysiology of preeclampsia remain incompletely understood, necessitating the exploration of preventive strategies [6].

Vitamin D, a fat-soluble vitamin that is essential for maintaining calcium and phosphorus homeostasis, has been postulated to play a role in the pathogenesis of preeclampsia [7]. Vitamin D deficiency, commonly encountered in pregnant women due to inadequate dietary intake and reduced sun exposure, has been associated with an increased risk of preeclampsia [8]. The immunomodulatory, anti-inflammatory, and vasculo-protective properties of vitamin D suggest that its supplementation during pregnancy may have potential benefits in preventing or attenuating the severity of preeclampsia [9,10].

Several observational and clinical studies have investigated the relationship between maternal vitamin D status and the risk of preeclampsia, yielding mixed results [11,12]. While some studies have reported an inverse association between serum 25-hydroxyvitamin D (25-OH-D) levels and the risk of preeclampsia, others have found no significant relationship [13]. Furthermore, the efficacy of vitamin D supplementation in early pregnancy as a preventive strategy for preeclampsia remains unclear, as randomized controlled trials have produced inconsistent findings [14,15].

A comprehensive synthesis of the available evidence is crucial for guiding clinical practice and informing public health policies. Systematic reviews and meta-analyses, which provide a rigorous and transparent approach to summarizing and appraising the existing literature, can help clarify the role of vitamin D supplementation in the prevention of preeclampsia [16,17]. To date, few systematic reviews have specifically addressed the effects of vitamin D supplementation during pregnancy on the risk of preeclampsia, although they have been limited by methodological issues such as not including studies where vitamin D supplementation began after the first trimester, studies where supplementation was discontinued, or studies that have not included the most recent evidence.

In light of the ongoing controversy surrounding the potential benefits of early pregnancy vitamin D supplementation for the prevention of preeclampsia, we conducted a systematic review of the literature. Our objective was to synthesize and critically appraise the available evidence from observational and interventional studies to determine the effects of early pregnancy vitamin D supplementation on the risk of preeclampsia. This review will provide a comprehensive and up-to-date summary of the current state of knowledge, contributing to a better understanding of the role of vitamin D in preeclampsia prevention and informing future research and clinical practice.

## 2. Materials and Methods

### 2.1. Review Protocol

The current study was carried out in March 2023, following a systematic review design by searching four electronic databases: Web of Science, Cochrane, PubMed, and Scopus. The review included literature published up to February 2023. The search strategy utilized medical subject headings (MeSH) keywords [18], such as “vitamin D”, “vitamin D deficiency”, “25-hydroxyvitamin”, “1,25(OH)2D3”, “pregnant women”, “gestation”, “25-hydroxycalciferol”, “25-hydroxyergocalciferol”, “pregnancy complications”, “(25-OH-D)”, and “preeclampsia”. The search was limited to English-language journal articles.

In adherence to the PRISMA criteria [19] and the PROSPERO guidelines [20], the study carried out a structured and systematic search protocol to identify relevant scientific papers examining the association between early vitamin D supplementation and preeclampsia. This systematic review was registered on the Open Science Framework (OSF) platform [21].

The aim of this systematic review was to comprehensively explore and address several research questions that shed light on the relationship between early vitamin D supplementation and preeclampsia. Vitamin D deficiency was considered for serum values of 25-OH-D below 30 ng/mL, while preeclampsia was defined as newly onset hypertension during pregnancy associated with end-organ dysfunction. The first research question aimed to determine the effects of early pregnancy nutritional supplementation with vitamin D on the incidence of preeclampsia and its complications. Secondly, this review sought to investigate whether any significant differences exist in pregnancy outcomes, such as preterm birth, intrauterine growth restriction, and neonatal complications, based on maternal vitamin D levels. Lastly, the review examined if there were any dose-dependent effects of vitamin D supplementation, which would help identify the optimal dose for the prevention or attenuation of preeclampsia and its associated adverse outcomes.

### 2.2. Selection Process

The main data sources for the compiled material encompassed the text, tables, figures, and supplementary online resources available within the articles. The selection process began with the removal of duplicate entries, followed by a meticulous evaluation of each abstract to assess its relevance to the research questions. Subsequently, a comprehensive review of the entire text was conducted for the remaining articles to ensure that they met the inclusion criteria. Moreover, an in-depth analysis of the reference lists of the gathered papers was performed by two independent researchers with the aim of identifying any pertinent literature that may have been overlooked during the initial search, thereby enhancing the comprehensiveness of this systematic review.

In this study, the main focus was on the following aspects: (1) characteristics of the study, including the number of studies, author, study location, the year the study was conducted, study design, and quality evaluation; (2) a summary of the findings, including patient count, mean or median age, gestational age, and infant weight; (3) vitamin D analysis among the included studies: amount of vitamin D intake, the onset of vitamin D supplementation, the threshold for vitamin D insufficiency, vitamin D levels, vitamin D insufficiency instances, occurrence of preeclampsia, maternal features, severity of preeclampsia, risk analysis, and unique attributes of the study.

The current review adopted a stringent set of criteria for a study’s inclusion in the final analysis. First, research is needed to investigate the link between vitamin D and preeclampsia. Second, it was imperative that the study discuss the initiation of vitamin D supplementation during the early stages of pregnancy. Third, a detailed account of the pregnancy outcomes was necessary. Lastly, all participants in the study must be aged 18 or older. The exclusion criteria comprised studies where vitamin D supplementation was initiated beyond the first trimester of pregnancy. Similarly, studies that failed to provide comprehensive data on patient demographics and medical backgrounds were not included. Studies that did not mention preeclampsia or did not assess the risk of preeclampsia in relation to vitamin D supplementation were also discarded. Case reports, literature reviews, meta-analyses, letters to editors, and short communications were not part of our selection.

### 2.3. Data Extraction and Quality Assessment

**Figure 1:**
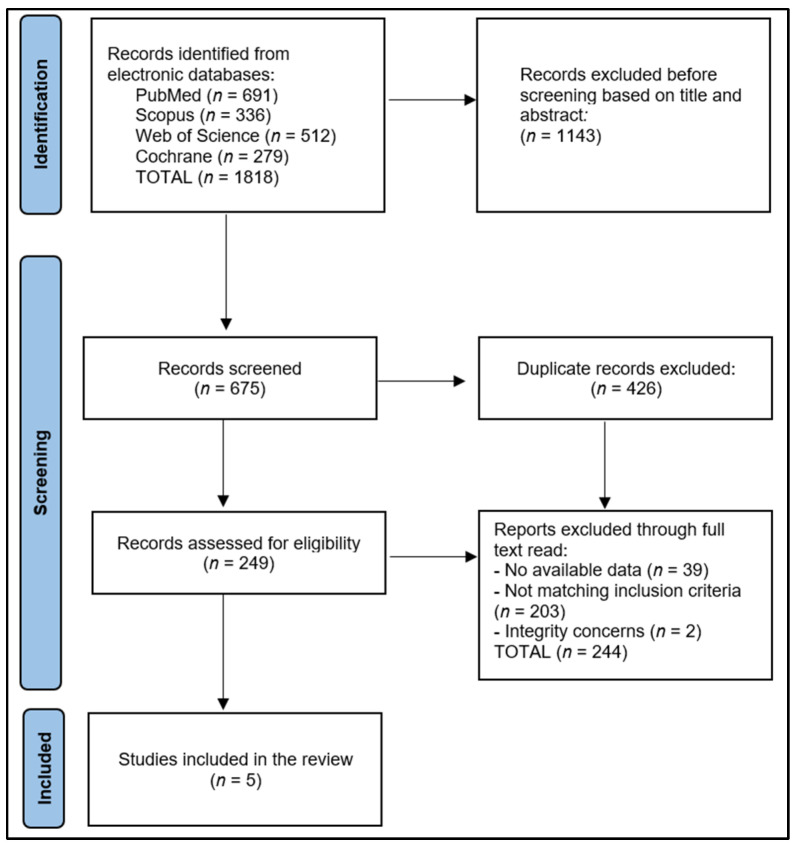
PRISMA flow diagram.

The initial exploration yielded 1818 studies, of which 426 were recognized as duplicates. After eliminating 1143 papers based on their abstracts, we scrutinized 249 full-text articles for relevance. Finally, five articles were eligible for inclusion in the systematic review, as shown in Figure 1. Utilizing the Study Quality Assessment Tools from the National Heart, Lung, and Blood Institute (NHLBI) [22], two investigators separately appraised the published works and recorded their conclusions. These instruments were customized to individual study designs, facilitating the identification of methodological or design issues.

We used the Quality Assessment Tool for Observational Cohort and Cross-Sectional Studies to evaluate the included studies. Each question within the tool received a score of 1 for “Yes” responses and 0 for “No” and “Other” responses in order to determine the final performance score. Research with scores from 0 to 4 was labeled as fair quality, those scoring between 5 and 9 as good quality, and those with a score of 10 or above were deemed excellent quality, as outlined in Table 1. To reduce selection bias, missing data, and measurement bias, two researchers independently assessed the quality of the chosen articles, thereby bolstering the reliability of the assessment process.

The analysis in Table 1 examined the characteristics of five studies that investigated the effects of early pregnancy vitamin D supplementation on preeclampsia risk. These studies spanned from 2013 to 2022, with the majority being conducted in Iran (two studies) [23,26], while one study each originated from India [24], the United States [25], and Romania [27]. All but one of the studies employed a randomized trial design, with the remaining study being a prospective study. In terms of study quality, one study was rated as excellent [25], one as good [24], and two as fair [23,26].

The predominance of randomized trials in this review provides a strong foundation for assessing the causality between early pregnancy vitamin D supplementation and preeclampsia risk. Randomized trials are considered the gold standard in evaluating the efficacy of interventions, as they reduce potential biases and allow for a more reliable comparison between the intervention and control groups. Furthermore, the overall good to excellent quality of the included studies lends credibility to the analysis, ensuring that the conclusions drawn from these studies are based on rigorously conducted research.

### 2.4. Assessment of Publication Bias

**Figure 2:**
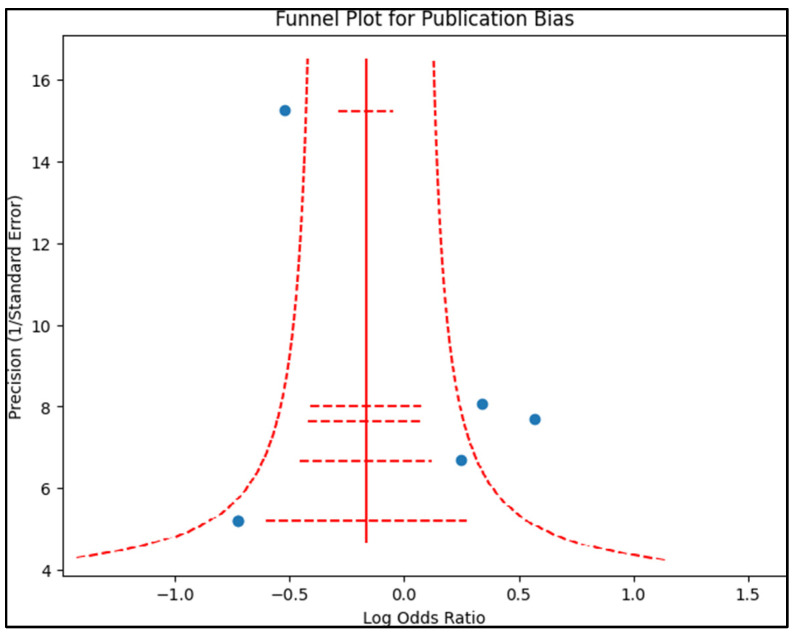
Funnel plot for publication bias.

Publication bias was examined by creating a funnel plot, where the standard error of the log odds ratio was plotted against its corresponding log odds ratio, as presented in Figure 2. The symmetry of the plot was visually examined and further assessed using Egger’s regression test and Begg’s test, with a *p*-value < 0.05 indicating significant publication bias. A sensitivity analysis was also conducted by removing one study at a time and recalculating the pooled odds ratios to evaluate the robustness of the results and examine the impact of individual studies on the overall effect size.

## 3. Results

The final analysis of this systematic review included a total of 1474 patients that were evaluated across the five studies. Table 2 describes the key demographic and clinical characteristics of all the analyzed patients. The studies demonstrate varying degrees of impact on gestational age, or SGA. In the study by Sablok et al. [24], there was a significant reduction in SGA in the vitamin D group (8.0%) compared to controls (19.2%). In contrast, study 1 [23] showed only slight differences between the groups, with 37.7% vs. 37.2% and 39.4% vs. 39.1%, respectively. Additionally, the studies by Mirzakhami and Dahma [25,27] reported minimal differences in gestational age or SGA outcomes between cases and controls. These variations suggest that the effect of vitamin D supplementation on gestational age, or SGA, may be influenced by factors such as dosage, timing, or other study-specific variables.

Infant weight also varied across the studies, such as in the research by Naghshineh et al. [23], where infants in the vitamin D group had a higher average weight (3.1 kg) compared to controls (2.8 kg). Sablok’s study [24] showed a modest increase in infant weight for the vitamin D group (2.6 kg) compared to controls (2.4 kg), while Dahma et al. [27] reported a similar trend, with 2.9 kg for cases and 2.7 kg for controls. The discrepancies in infant weight outcomes among the studies may be attributed to differences in vitamin D dosage, the timing of supplementation, or other factors that were not controlled for in the studies.

Also, vitamin D intake varied considerably across the studies, ranging from 600 IU/day to 50,000 IU every two weeks. Studies 1 and 3 [23,25] used daily supplementation, while Sablock et al. [24] employed biweekly doses. Furthermore, the same study had a unique dosing regimen, administering a single dose of either 60,000 IU or 120,000 IU between 14 and 20 weeks. It is possible that the varying vitamin D dosages contributed to the inconsistencies in the outcomes observed across the studies, highlighting the need for further research to determine the most effective dosage for reducing the risk of preeclampsia.

Lastly, the timing of supplementation initiation also differed among the studies. The beginning of supplementation ranged from the first trimester to 20 weeks of pregnancy, with some studies specifying exact week ranges, such as the study by Mirzakhani et al. [25] starting between weeks 10–18, and others providing broader timeframes. These differences in the timing of supplementation may have also contributed to the inconsistencies in the observed outcomes, as the optimal time to begin vitamin D supplementation for the prevention of preeclampsia remains unclear.

Table 3 presents an evaluation of vitamin D levels, vitamin D insufficiency, preeclampsia rates, and maternal features in the included studies of the systematic review. The vitamin D insufficiency threshold varied across studies, ranging from less than 20 ng/mL to less than 32 ng/mL. This variability in defining vitamin D insufficiency should be taken into consideration when comparing results between studies, as it may influence the observed associations between vitamin D levels and preeclampsia risk. Vitamin D levels were reported in five studies, with cases generally showing lower levels than controls. This observation suggests a possible link between lower vitamin D levels and an increased risk of preeclampsia.

Vitamin D insufficiency percentages were reported in six studies, with a wide range of prevalence (from 22.6% to 78.0%). This variation may be attributed to differences in the study populations, geographical locations, and the aforementioned variability in defining vitamin D insufficiency. In most studies, the prevalence of preeclampsia was lower in cases (those receiving vitamin D supplementation) compared to controls, supporting the potential role of vitamin D supplementation in reducing preeclampsia risk. Furthermore, maternal features, such as parity and baseline systolic blood pressure (SBP), were reported in some studies, providing additional context for interpreting the results. For example, nulliparous women (100% of participants) in studies 1, 2, and 3 may be at a higher risk of preeclampsia compared to multigravida women (100% of participants in study 4). Similarly, differences in baseline SBP between cases and controls, as reported in studies 4 and 5, could be potential confounding factors in the relationship between vitamin D supplementation and preeclampsia risk.

In Sablock’s study [25], the cases receiving vitamin D supplementation had lower vitamin D levels than the controls, with levels of 17.1 vs. 34.9 ng/dL, 18.5 vs. 20.6 ng/dL, and 13.1 vs. 16.2 ng/dL, respectively. In contrast, the study by Mirzakhani et al. [25] showed higher vitamin D levels in cases compared to controls (19.7 vs. 24.4 ng/dL), while Dahma et al. [27] reported substantially higher vitamin D levels in cases compared to controls (32.3 vs. 22.5 ng/dL). These differences in vitamin D levels between cases and controls across the studies may be attributable to the variation in vitamin D supplementation dosages and timing, as well as potential differences in baseline vitamin D levels or sunlight exposure.

The percentage of patients who developed preeclampsia in the cases and control groups varied across the studies. In general, the case group (those receiving vitamin D supplementation) consistently demonstrated a lower percentage of preeclampsia compared to the control group. For example, the preeclampsia percentages in cases vs. controls were 2.9% vs. 7.0% in study 1, 11.1% vs. 21.1% in study 2, 14.9% vs. 30.9% in study 3 [25], and 15.7% vs. 30.6% in Sasan’s study [26]. However, in Dahma’s study [27], the difference in preeclampsia percentage between cases and controls was smaller (14.8% vs. 18.6%).

Overall, the studies showed mixed results in terms of the effect of vitamin D supplementation on preeclampsia severity, with some studies reporting significant reductions in risk while others showed non-significant or even increased risk, as described in Table 4. Two studies [23,25] reported a reduced risk of preeclampsia in the vitamin D supplementation group, with ORs ranging from 0.26 to 0.31. Among these, the study by Mirzakhani et al. [25] had significant results (*p* = 0.040), while in study 1 [23], the authors did not reach statistical significance (*p* = 0.105). In contrast, other studies found a higher risk of preeclampsia in patients with insufficient vitamin D levels, with ORs of 4.60, 1.94, and 2.52 [24,26,27], all of which were statistically significant (*p* < 0.001).

The severity of preeclampsia was reported in only one study [23], with 43% of severe preeclampsia developed by patients without vitamin D supplementation, compared to 0% among those who followed the supplementation protocol of 600 UI/day. In terms of secondary outcomes, several studies reported significant findings, such as Naghshineh et al. [23], who demonstrated a significant reduction in preterm labor (*p* = 0.006) among those receiving vitamin D supplementation, while Sablok et al. [24] found a significant decrease in the incidence of APGAR scores < 7 (*p* < 0.001) and higher vitamin D levels in cord blood (*p* < 0.001) in the supplementation group. Study 5 showed that vitamin D levels of 30 ng/mL or higher at trial entry and in late pregnancy were associated with a lower risk of preeclampsia.

## 4. Discussion

### 4.1. Summary and Contributions

This systematic review aimed to investigate the effects of early pregnancy vitamin D supplementation on the incidence and severity of preeclampsia and explore its impact on pregnancy outcomes. The findings suggest that early vitamin D supplementation may play a role in reducing the risk of preeclampsia, though the extent of this relationship remains unclear. The heterogeneity in study designs, participant characteristics, and doses of vitamin D supplementation used in the included studies contributed to the complexity of the results. Furthermore, though some studies observed improvements in pregnancy outcomes with early vitamin D supplementation, the evidence remains inconclusive, warranting further investigation.

The review found that several studies [24,25,26,27] reported a significant reduction in preeclampsia risk among women who received early vitamin D supplementation. However, the specific dose and duration of vitamin D supplementation varied across the studies from 400 IU/day to 50,000 IU every two weeks, or a single dose of 120,000 units, making it difficult to establish a definitive relationship. Additionally, differences in participant characteristics, such as baseline vitamin D status and preexisting risk factors for preeclampsia, may have influenced the results. Based on the available data, we suggest initiating supplementation up to the 20th week of pregnancy, regardless of whether it is continued until delivery or not, with a dose of approximately 25,000 IU/week. However, with weekly administration, it may be necessary to monitor calcemia and calciuria as potential markers of vitamin D overdose. Nevertheless, while some studies reported benefits with specific doses, others found no significant dose-dependent effects. The lack of consistent findings may be due to differences in study designs, participant characteristics, and outcome measures. In high doses, vitamin D may be effective in preventing preeclampsia, given its accumulation in adipose tissue. Further investigation is warranted to determine the optimal dosing regimen during pregnancy, such as daily, weekly, or a single administration.

The timing of vitamin D administration and the attainment of optimal vitamin D levels are crucial factors to consider. There is substantial immunologic evidence, early biomarker detection, and signs of abnormal placental development and function suggesting that preeclampsia is a disorder originating from early placentation [28,29]. As such, the immunomodulatory effects of vitamin D during pregnancy have been studied in recent years, observing an important effect on regulatory T cells that are essential during pregnancy in preventing autoimmune diseases [30]. Adequate vitamin D concentrations must be present during implantation and placentation, as these two stages involve significant maternal-fetal immune interactions that play a critical role in preeclampsia [31,32]. A notable impact of vitamin D3 on human extravillous trophoblast invasion in early pregnancy was observed at a concentration of 40 ng/mL [33].

Although there appears to be no association between vitamin D and preeclampsia by maternal age, one meta-analysis included pregnant women only in the 20–34 age range [34]. Additionally, the included randomized controlled trials lack information on the achieved vitamin D serum levels. Consequently, it remains uncertain whether the benefits of vitamin D supplementation are greater for women with persistent deficiency or for those attaining optimal serum vitamin D levels. Nonetheless, the primary objective of our study was to assess whether early (<20 weeks of gestation) clinical vitamin D supplementation itself could reduce the incidence of a clinically significant outcome such as preeclampsia, and our findings support this hypothesis, reporting significant odds ratios of the protective role of early vitamin D supplementation (OR = 0.28, *p* = 0.040) [25].

One finding might suggest that a circulating vitamin D level of around 40 ng/mL during pregnancy may reduce the risk of preeclampsia associated with vitamin D deficiency. This observation aligns with the conclusion of the NICHD trial, which suggests that a circulating 25-OH-D level of 40 ng/mL is necessary for optimal 1,25(OH)2D production during pregnancy [35,36]. In the NICHD trial, participants were given 4000 IU, 2000 IU, or 400 IU of vitamin D after 12 to 16 weeks of gestation, with the 4000 IU dosage exhibiting the most significant impact on decreasing the composite rate of pregnancy comorbidities.

The VDAART trial [34] revealed the presence of distinct biological processes consistent with preeclampsia as early as the tenth week of pregnancy in women with low vitamin D status who later developed preeclampsia. This contrasted with the observations in vitamin D that sufficiently matched controls. These findings suggest the development of systematic immunologic changes at the earliest placental stage and before enrollment in the VDAART. In comparison, participants with adequate vitamin D levels at the trial’s onset (21.8%) and who maintained this status throughout late pregnancy exhibited a significantly reduced risk of preeclampsia. While vitamin D is implicated in preeclampsia’s pathophysiology, its role could be mediated by other interacting factors, implying that a subgroup of pregnant women with low vitamin D status may derive the most significant benefits from supplementation. Moreover, it was previously suggested that other interacting factors, such as nulliparity, are a moderate risk factor for the development of preeclampsia, thus the necessity to determine vitamin D levels and provide nutritional supplementation to pregnant women found at risk [35].

Moreover, the NICHD trial revealed a significant risk of hypertensive pregnancy disorders, including preeclampsia, after adjusting for race (*p* = 0.05) across the different treatment arms that ranged between 400 IU and 4000 IU of vitamin D [36]. This finding is in line with the results of another recent trial that investigated vitamin D supplementation with a single dose of 60,000–480,000 IU determined by a serum vitamin D level higher than 20 ng/mL, between 10–20 ng/mL, and lower than 10 ng/mL, where almost half of the patients had vitamin D levels below 10 ng/mL after 20 weeks of pregnancy [37]. Similar to the NICHD trial, no adverse events related to vitamin D3 supplementation were observed [37]. These findings suggest that the current criterion for vitamin D deficiency (<20 ng/mL) might not be adequate for preventing pregnancy-related adverse events such as preeclampsia.

It is worth noting that the 4000 IU dose of vitamin D3 in the VDAART only led to sufficient serum vitamin D levels (≥30 ng/mL) in 74% of pregnancies at 32 to 38 weeks of gestation. This percentage is lower than the 82% observed one month before delivery in the NICHD trial using a similar dosage [24]. This discrepancy highlights the importance of tailoring vitamin D supplementation to individual needs and monitoring serum levels to ensure optimal outcomes in pregnancy.

Our observations are consistent with the contemporary understanding that preeclampsia stems from a preclinical phase of abnormal placentation during early pregnancy [28]. The data imply that earlier supplementation with vitamin D, potentially even prior to embryo implantation, could be necessary to improve pregnant women’s vitamin D status and prevent preeclampsia. The hypothesis of vitamin D supplementation during pregnancy should be further explored through larger clinical trials to determine the potential benefits of earlier vitamin D supplementation in pregnancy. Nevertheless, although vitamin D supplementation is generally well tolerated, particularly within recommended dosages, potential adverse effects can arise from excessive intake, causing vitamin D toxicity associated with hypercalcemia, nausea, vomiting, weakness, and frequent urination [38]. More severe complications may include kidney stones, kidney injury, and organ or soft tissue calcification. During pregnancy, considerations are magnified due to potential impacts on both mother and fetus; excessive maternal vitamin D levels may lead to fetal hypercalcemia, potentially inciting heart and kidney complications [39]. Some literature also suggests a correlation between elevated maternal vitamin D and an increased risk of offspring food allergies within the first two years of life.

The review identified limited evidence on the optimal dose of vitamin D supplementation for the prevention or attenuation of preeclampsia and its associated adverse outcomes, although early supplementation was a piece of clear evidence for preeclampsia prevention. Furthermore, our positive results may potentially underestimate the prophylactic effect of vitamin D supplementation. This is because the majority of the study participants were not stringently selected based on their baseline circulating vitamin D levels or their attainment of optimal vitamin D concentrations following supplementation. Thus, additional research with a focus on these factors may reveal an even more pronounced effect of vitamin D supplementation on preventing preeclampsia.

### 4.2. Study Limitations and Future Perspectives

This systematic review was conducted using a robust search strategy and rigorous inclusion and exclusion criteria, enhancing its comprehensiveness and validity. However, some limitations should be acknowledged. The small number of included studies and their methodological heterogeneity limited the ability to draw definitive conclusions. Additionally, the review was restricted to English-language articles, which may have excluded relevant research published in other languages. The reliance on published literature also raises the possibility of publication bias, as studies with null or negative findings are less likely to be published. Nevertheless, a publication bias analysis was performed to acknowledge this risk.

It is important to note, however, that the majority of the studies were conducted in Iran, which may limit the generalizability of the findings to other populations. Cultural, genetic, and environmental factors may influence the relationship between vitamin D supplementation and preeclampsia risk, and therefore it would be beneficial to include more studies from diverse geographic regions in future research. Furthermore, the lack of standardized reporting on preeclampsia severity and the absence of a common definition for vitamin D sufficiency limit the comparability of the studies.

The findings of this systematic review highlight the need for further research on the relationship between early vitamin D supplementation and preeclampsia as well as its impact on pregnancy outcomes. Future studies should focus on conducting well-designed randomized controlled trials with standardized vitamin D supplementation regimens and diverse populations to improve the generalizability of the results. Additionally, research should aim to identify the optimal dose of vitamin D supplementation to prevent or mitigate preeclampsia and its associated adverse outcomes. In the interim, healthcare providers should consider individual patient characteristics and risk factors when recommending vitamin D supplementation during pregnancy.

## 5. Conclusions

The analysis of selected studies encompassing 1594 patients revealed mixed results, with some studies demonstrating a significantly reduced preeclampsia risk associated with vitamin D supplementation while others showed no significant difference. The discrepancies in outcomes may be attributed to variations in vitamin D dosages, the timing of supplementation, baseline vitamin D levels, sunlight exposure, and other study-specific factors. Moreover, inconsistencies in defining vitamin D insufficiency and diverse secondary outcomes were observed across the studies. Although the current evidence suggests a potential role for vitamin D supplementation in reducing the risk of preeclampsia, further research is needed to identify the optimal dosage and timing of supplementation for preeclampsia prevention or attenuation.

## Figures and Tables

**Table 1:** Study characteristics.

Study and Author	Country	Study Year	Study Design	Study Quality
1 [23] Naghshineh et al.	Iran	2013	Randomized Trial	Fair
2 [24] Sablock et al.	India	2015	Randomized Trial	Good
3 [25] Mirzakhani et al.	USA	2016	Randomized Trial	Excellent
4 [26] Sasan et al.	Iran	2017	Randomized Trial	Fair
5 [27] Dahma et al.	Romania	2022	Prospective Study	Good

**Table 2:** Summary of findings in the included studies.

Study Number	Patients (Cases vs. Controls)	Average Age (Cases vs. Controls)	Gestational Age or SGA (Cases vs. Controls)	Infant Weight (kg) (Cases vs. Controls)	Vitamin D Intake (IU)	Beginning of Supplementation
1 [23] Naghshineh et al.	138 (68 vs. 70)	25.0 vs. 24.8	37.7% vs. 37.2%	3.1 vs. 2.8	600/day	<16 weeks
2 [24] Sablok et al.	180 (120 vs. 60)	NR	8.0% vs. 19.2%	2.6 vs. 2.4	one dose of 60,000 or one dose of 120,000	one dose between 14–20 weeks
3 [25] Mirzakhani et al.	816 (408 vs. 408)	27.5 vs. 27.2	38.7% vs. 38.8%	NR	4400/day vs. 400/day	Weeks 10–18
4 [26] Sasan et al.	142 (70 vs. 72)	32.0 vs. 29.8	NR	NR	50,000/two weeks	14 weeks
5 [27] Dahma et al.	198 (139 vs. 59)	NR	NR	2.9 vs. 2.7	4000/day vs. 2000/day	First trimester

NR—not reported; IU—international units; cases—received vitamin D, controls—did not receive vitamin D supplementation; SGA—small for gestational age (at delivery).

**Table 3:** Evaluation of vitamin D levels, preeclampsia, and maternal features in the included studies.

Study	Vitamin D Insufficiency Threshold	Vitamin D Levels (Cases vs. Controls) ng/dL	Vitamin D Insufficiency (%)	Preeclampsia (Cases vs. Controls)	Maternal Features
1 [23]	<32 ng/mL	NR	NR	2.9% vs. 7.0%	Nulliparous—100%; No sign of vitamin D deficiency
2 [24]	<20 ng/mL	18.5 vs. 20.6	77.5%	11.1% vs. 21.1%	Primigravida—100%
3 [25]	<30 ng/mL	19.7 vs. 24.4 *	29.2%	14.9% vs. 30.9%	Nulliparous—100%
4 [26]	<25 ng/mL	NR	78.0%	15.7% vs. 30.6%	Multigravida—100% (average three previous pregnancies) Baseline SBP 115.9 mm/Hg vs. 114.5 mm/Hg
5 [27]	<30 ng/mL	32.3 vs. 22.5 *	22.6% vs. 55.8%	14.8% vs. 18.6%	Baseline SBP 128.5 mm/Hg vs. 141.4 mm/Hg Parity (>3) 11.5% vs. 15.3%

*—significant differences at *p* < 0.05; NR—not reported; cases—received vitamin D, controls—did not receive vitamin D supplementation.

**Table 4:** Evaluation of outcomes.

Study Number	HR/OR	Particularities (Cases vs. Controls)
1 [23] Naghshineh et al.	0.26 (*p* = 0.105)	Preterm labor—6.0% vs. 24.3% (*p* = 0.006)
2 [24] Sablock et al.	4.60 * (*p* < 0.001)	Preterm labor—8.3% vs. 21.1% (*p* = 0.080) APGAR < 7—1.1% vs. 13.0% (*p* < 0.001) Vitamin D in cord blood—22.7 vs. 17.3 (*p* < 0.001)
3 [25] Mirzakhani et al.	0.28 (*p* = 0.040)	Vitamin D levels of 30 ng/mL or higher at trial entry and in late pregnancy were associated with a lower risk of preeclampsia
4 [26] Sasan et al.	1.94 * (*p* < 0.001)	24 h proteinuria (mg/cc) 132.2 vs. 154.9 BMI > 30 kg/m^2^—18.6% vs. 29.6%
5 [27] Dahma et al.	2.52 * (*p* < 0.001)	Parity (>2) carried a 1.89 higher risk for preeclampsia

*—reference for patients with insufficient vitamin D levels; NR—not reported; cases—received vitamin D, controls—did not receive vitamin D supplementation; HDL—High-density lipoprotein; APGAR – activity, pulse, grimace, appearance, and respiration; BMI—body mass index.

## Data Availability

Not applicable.
